# Recent Advances in Asymmetric Wettability Dressings for Wound Exudate Management

**DOI:** 10.34133/research.0591

**Published:** 2025-01-14

**Authors:** Fang Wang, Wenqing He, Bing Dai, Xueji Zhang, Yongqiang Wen

**Affiliations:** ^1^ Guangdong Laboratory of Artificial Intelligence and Digital Economy (SZ), School of Biomedical Engineering, Health Science Center, Shenzhen University, Shenzhen 518060, P. R. China.; ^2^ School of Chemistry and Biological Engineering, University of Science and Technology Beijing, Beijing 100083, P. R. China.

## Abstract

The management of wound exudate is of vital importance for wound healing. Exudate accumulation around wound prolongs inflammation and hinders healing. Although traditional dressings can absorb wound exudate, they are unable to drain exudate in time, often resulting in a poor feature with wound healing. In recent years, the appearance of asymmetric wettability dressings has shown great potential in exudate management. Here, we summarize the latest progress of 3 kinds of asymmetric wettability wound dressings in exudate management, including Janus structure, sandwich structure, and gradient structure. The most common Janus structural dressing among asymmetric wettability dressings is highlighted from 2 aspects: single-layer modified Janus structure and double-layer Janus structure. The challenges faced by asymmetric wettability wound dressings are discussed, and the developing trends of smart wound dressings in this field are prospected.

## Introduction

Skin is the largest organ and the human body’s first immune barrier, playing a vital role in many physiological activities [1–4]. Although skin has the ability of self-healing, the healing process is often affected when large area of skin defects accompanied with heavy bleeding or infection caused by the underlying diseases such as diabetes mellitus [5,6]. Wound healing is a delicate and complex process that often goes through several stages: inflammation, proliferation, and remodeling [7,8]. Prolonged inflammatory period is an important factor leading to the difficulty of wound healing, especially for chronic wound [9,10], while excessive exudate around chronic wound will make the wound overhydrate, inhibit fibroblast proliferation, and prolong the inflammatory period, thus hindering the healing process [11]. Therefore, effective management of wound exudate is urgent. Traditional dressings for exudate management (such as gauze and cotton) have good water absorption and retention ability, but their functions are relatively simple [12]. After a long period of use, the exudate remaining in the dressings may reverse permeate into the wound to hinder healing. Especially, it is easy to absorb the exudate and stick to the wound, which may cause secondary injury to the wound without replacement in time [13,14]. Similarly, hydrophobic dressings can effectively prevent the growth of bacteria due to the anti-adhesion property, but their low fluid absorption and the exudate accumulation near the wound are difficult to provide a moist environment for wound healing, affecting the healing speed [15]. It can be seen that a single-function wettability material is usually not effective in removing wound exudate and helping wound healing.

For the past few years, development of infiltration field provides lots of new ideas and new methods for the study of liquid transport [16–23]. Especially, the emergence of asymmetrical wettability structure has shown great potential in wound exudate management [24–26]. In contrast to homogeneous materials, materials with asymmetric wettability can follow a wettability gradient and autonomously move the liquid from one end to the other, and effectively prevent a large amount of liquid backflow [27–30]. Usually, the 2 sides of this structure are hydrophilic and hydrophobic surfaces. The hydrophobic layer acts as a dense layer of water-resistant and antibacterial to block the attack of foreign bacteria and control the gas–liquid exchange of the wound to prevent wound dehydration. The hydrophilic layer is mostly a breathable porous layer that absorbs wound exudate and supports cell adhesion, migration, and proliferation [31,32]. The porous structure is also conducive to load anti-inflammatory and antibacterial drugs or bioactive factors promoting healing or other therapeutic effects, and plays a synergistic role in wound healing [33]. The effective combination of the 2 layers can achieve the balance of permeability and hydrophobicity of the wound dressings, and directionally and timely discharge the excess exudate in the wound, creating a suitable environment for wound healing [34]. It can be seen that multifunctional wound dressings combined with efficient exudate management is an urgent need to build up the microenvironment of wound and promote healing.

**Fig. 1.**
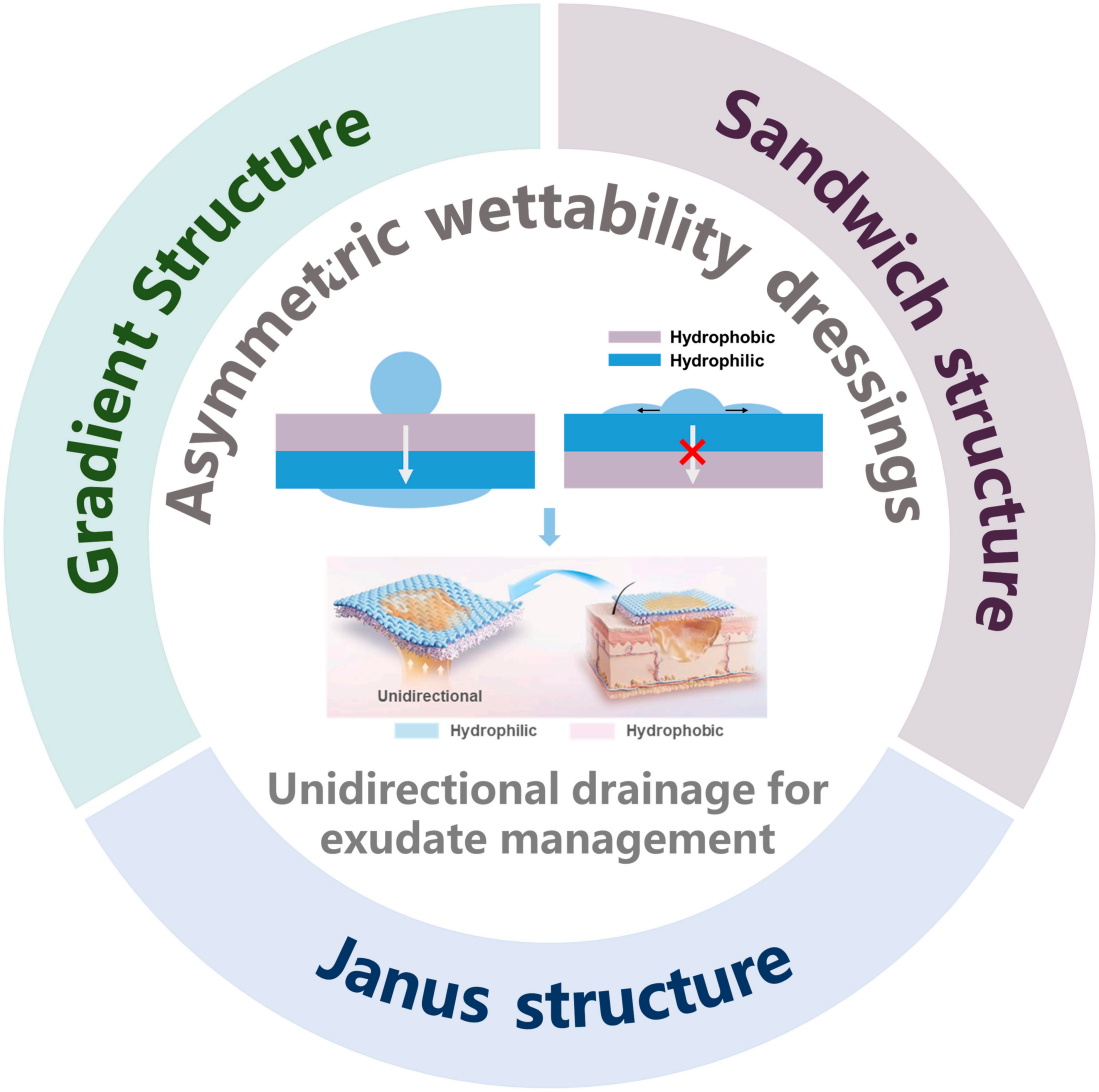
Schematic diagram of the asymmetric wettability dressings for exudate management.

Here, we discussed the limitations of traditional dressings in wound exudate management and the necessity of exudate management dressings, and proposed the advantages of asymmetric wettability wound dressings and challenges in overcoming these limitations. Then, the recent progress of asymmetric wettability dressings in the treatment of wound exudate, including Janus structure, sandwich structure, and gradient structure, was summarized (Fig. 1). The most common Janus structural dressing among asymmetric wettability dressings was highlighted from 2 aspects: single-layer modified Janus structure and double-layer Janus structure. Finally, we prospected the demands and development trends of smart wound dressings from the viewpoint of real-time monitoring and analysis of wound condition.

## Importance of Exudate Management Dressing

Wound exudate was a natural and essential part of wound healing, which not only prevented wound from drying out but also helped with cell migration and tissue repair and provided essential nutrients and growth factors for wound healing [35,36]. However, during the healing process of chronic wounds, body fluids, cells, and bacteria formed a large amount of liquid around wound, which would prolong the inflammatory period and was not conducive to wound healing [37,38]. This was because the leakage of excessive exudate provided a way for microorganisms to enter the wound, causing infection and producing odors. The leakage of exudate might also cause the skin around the wound to macerate and erode, expanding the area of injury and increasing the difficulty of wound healing [39]. Through effective management, the harmful effects of wound exudates could be greatly reduced [40,41]. Traditional dressing usually focused on the absorption of exudate, resulting in frequent dressing replacement, increasing the double burden of patients and medical staff [42–45]. Therefore, there was an urgent need to design a new dressing that could not only treat excessive wound exudate by absorbing or evaporating it from the surface of dressing but also maintain a wet environment, promoting granulation tissue growth and preventing infection.

## Classification of Asymmetric Wettability Dressings for Exudate Management

In the past few years, asymmetric wettability materials had been actively studied and applied in the field of wound dressings [46]. Compared to homogeneous materials, asymmetrical wettability materials with the directional fluid transport capabilities could spontaneously transfer fluid from one side to another side following a wettability gradient [47–49], showing great potential in wound exudate management. We classified the structural types of asymmetric dressings and summarized the research progress of different forms of wound dressings in exudate management in recent years.

### Janus structure

Janus interface materials were 2-dimensional material with asymmetric properties on each side, in which Janus membrane was a special member [50]. According to the membrane configuration, Janus membrane could be divided into 2 types. Broadly speaking, the 2 sides of Janus membrane should have different properties, such as composition, surface charge, and wettability. However, in a narrow sense, the definition of Janus membrane should be more strictly limited to membranes with opposite properties on both sides. The contrary properties just like hydrophilic/hydrophobic and positive/negative charge could be achieved through chemical or physical modifications [51]. The rise of Janus membranes had brought new opportunities for wound dressings and revealed new functions that traditional wound dressings could not achieve.

Janus structure dressing was one of the most studied and common wound dressings with wettability asymmetric. There were usually 2 types, including asymmetric modification to get one material with 2 sides of different wettability or asymmetric fabrication to obtain different materials with different wettability. This asymmetry could provide an intrinsic driving force causing the liquid to move in a specified direction [22]. By combining a hydrophobic impermeable layer with a hydrophilic breathable layer, Janus wound dressing guided wound exudate to spontaneously transfer from wound to the outside, enabling effective, on-demand management of wound exudate [52]. In recent years, researchers had used the unique properties of Janus membranes for directional fluid delivery to design a series of advanced smart wound dressings.

#### Single-layer modified Janus structure

**Fig. 2.**
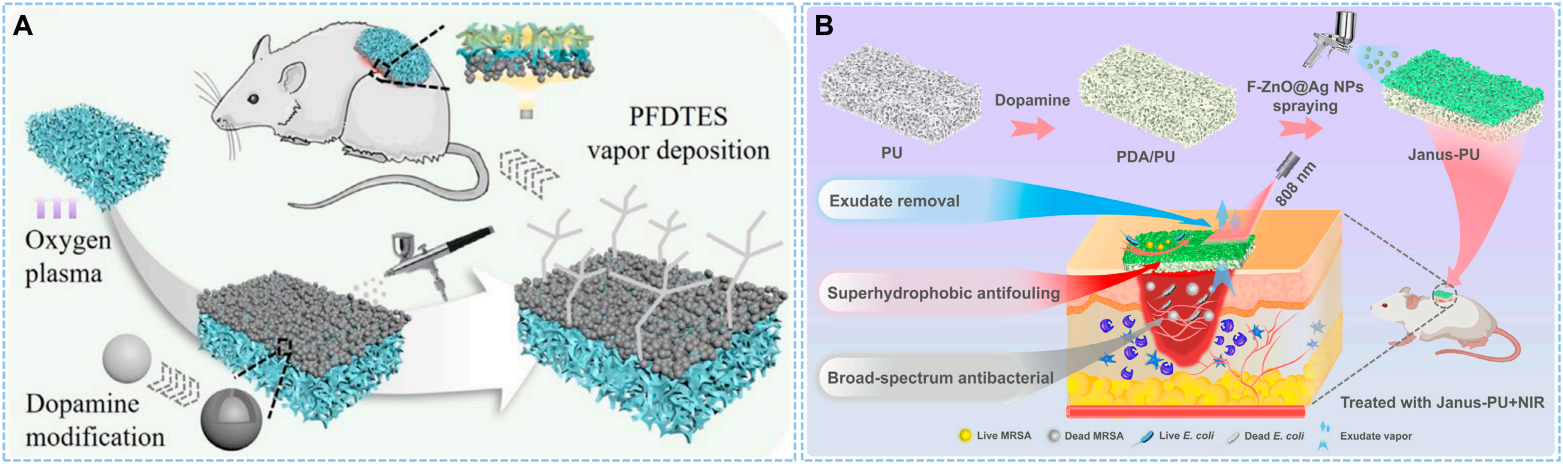
Single-layer modified asymmetric wettability dressing with Janus structure. (A) Schematic illustration of the preparation of Janus sponge dressing. Reproduced with permission [53]. Copyright 2021, Elsevier*.* (B) Schematic representation for construction of multifunctional Janus dressing and the process of wound healing effect under NIR irradiation. Reproduced with permission [54]. Copyright 2023, Elsevier*.*

Single-layer modified asymmetric wettability dressing usually referred to the preparation of a uniform single film by electrospinning, freeze-drying technology, or other methods, firstly, and then modifying one side of the film with the hydrophobic or hydrophilic materials to finally obtain the asymmetric wettability dressing. Zhao and colleagues [53] constructed a novel Janus polyurethane (PU) sponge dressing, in which an asymmetric wettability structure was obtained by spraying polydopamine (PDA)-modified silica particles onto one surface of the PU sponge. The superhydrophilic material provided enough drainage power allowing liquid to travel one way without wetting the superhydrophobic layer, solving the challenge of overhydration between dressing and wound (Fig. 2A). Further, Zhang’s group exploited a multifunctional dressing by coating one side of a hydrophilic PU sponge with near-infrared (NIR) responsive superhydrophobic nanoparticles. The one-way discharge of wound exudate was controlled by NIR irradiation, creating the suitable wetting environment for wound healing (Fig. 2B) [54].

#### Double-layer Janus structure

**Fig. 3.**
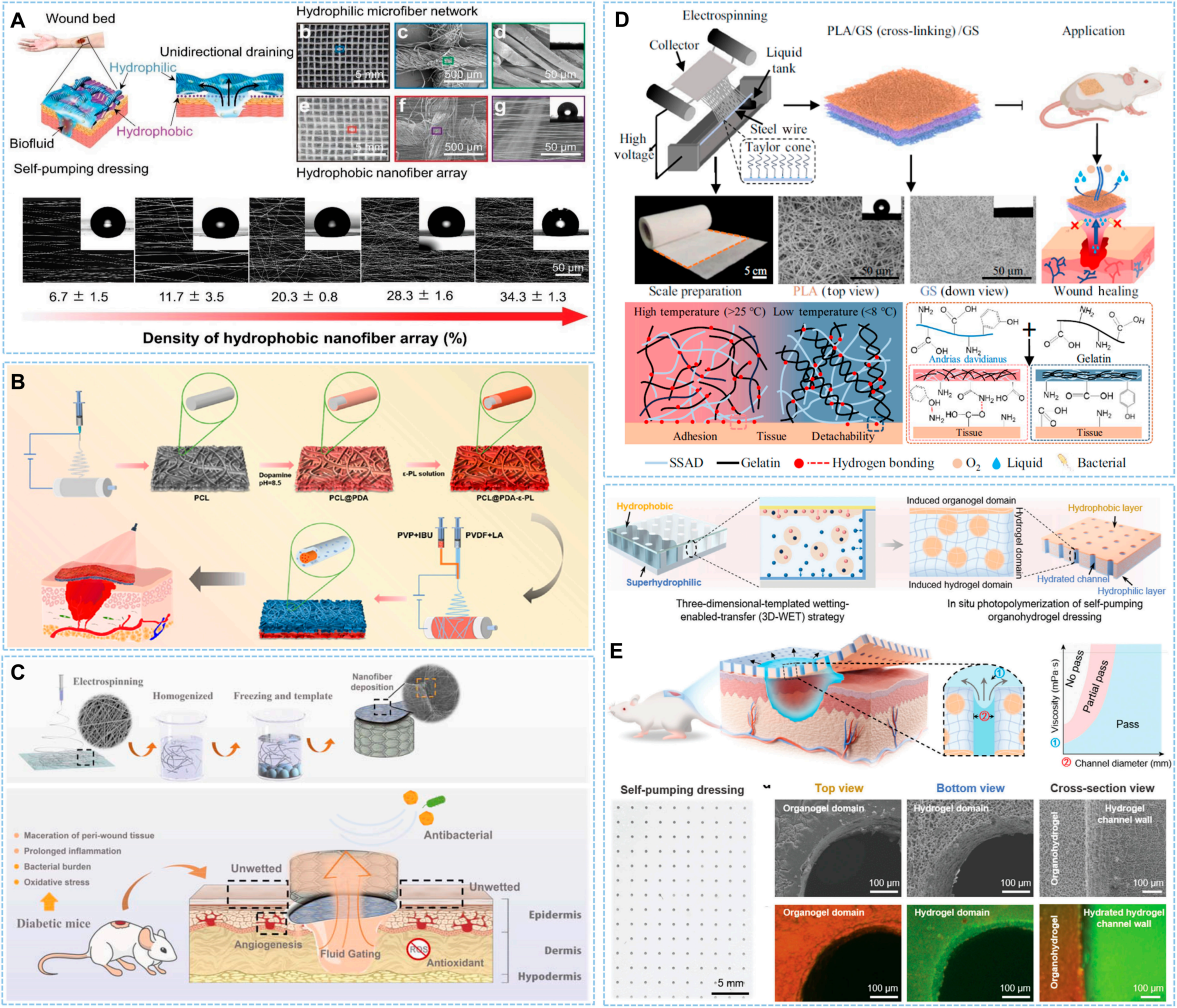
Double-layer asymmetric wettability dressing with Janus structure. (A) Schematic representation of preparing a self-pumping dressing and the process in wound exudate draining. Reproduced with permission [46]. Copyright 2018, Wiley-VCH. (B) Schematic illustration of fabricating a PCL @ PDA-ε-PL nanofiber membrane and the property of directional water transport. Reproduced with permission [62]. Copyright 2023, American Chemical Society. (C) Schematic illustration of constructing the nanofibrous aerogel and the wound therapy process. Reproduced with permission [56]. Copyright 2021, Elsevier. (D) Schematic illustration of the construction and application of temperature-responsive detachable nanofiber dressing. Reproduced with permission [69]. Copyright 2024, Springer Nature. (E) Schematic diagram of the preparation of self-pumping organohydrogel dressing. Reproduced with permission [71]. Copyright 2024, Wiley-VCH.

According to the material characteristics and production methods, wound dressings could be divided into various forms, including textile-based [55], micro/nanofibrous-based [56], hydrogel-based [57], and sponge/foam-based dressing [53], among which electrospinning nanofibers had been studied extensively and deeply due to their similarity to the structure and composition of natural extracellular matrix [58,59]. In addition, the permeability of wound dressing directly affected wound healing because an air-tight dressing might cause the wound to be starved of oxygen, increasing the risk of infection and even causing tissue necrosis. The nanofibers with the characteristics of adjustable permeability and large specific surface area could improve the uptake of nutrients and oxygen, which was beneficial to wound repair [60,61]. Wang and colleagues [46] constructed a self-pumping dressing to deposit a PU nanofiber array on hydrophilic cotton gauze that could transport biological fluid one way, preventing excess biological fluid from wetting wound, allowing wound to heal faster than traditional dressings (Fig. 3A). Premature leakage due to drug attachment to the outer surface of the fiber membrane was a common problem. Gao and colleagues [62] deposited nanofibers on hydrophilic poly(ε-caprolactone) (PCL) @ PDA membrane by coaxial electrospinning technology to prepare an asymmetric wettability nanofiber dressing with one-way liquid delivery. The anti-inflammatory drug was wrapped in core layer and controlled release by lauric acid with a melting point of 43 °C, which could effectively prevent premature leakage (Fig. 3B). Chronic wound was often accompanied by tissue edema and large amounts of exudate, while the inherent hydrophilicity of traditional cotton layer was limited for wound with excess exudate. Therefore, dressings with more directional fluid delivery capacity were needed to absorb large amount of exudate [63,64]. To enhance the transfer efficiency and absorption rate of wound exudate, Wen and colleagues [56] developed a Janus nanofibrous aerogel that could transfer liquid independently and unidirectionally. What is more, the Janus nanofibrous aerogel could absorb a large amount of liquid without reflux, showing great potential in the therapy of diabetic wound (Fig. 3C). In addition, evaporation caused by the photothermal effect could promote the discharge of exudate from dressing so that the dressing remained unsaturated. Chen and colleagues [65] prepared an asymmetric wettability dressing to promote spontaneous transfer of liquid along with hydrophobic side to hydrophilic layer by the simulation of lotus leaves. The photothermal effect generated by the addition of PDA in the hydrophilic layer helped exudate from dressing, which solved the problem of excessive hydration of the dressing on wet wound, delaying healing.

Janus dressing typically consisted of different materials with low interfacial affinity between adjacent layers, resulting in easy separation between layers, especially when affected by complex external conditions. To this end, Huang and colleagues [66] developed a multifunctional Janus wound dressing that used a single poly (lactide caprolactone) (PLCL) for both the hydrophilic layer and the hydrophobic layer. The hydrophobic fiber was transformed into hydrophilic fiber through the hydrolysis reaction to enhance the interface affinity between the hydrophilic/hydrophobic PLCL fibers, which had high external force resistance and continuous unidirectional exudate transfer capability.

After absorbing exudate, dressing could easily adhere to wound through scab or new granulation tissue, resulting in serious wound avulsion during dressing replacement, which brought great pain to patients. Therefore, the ideal dressing should not only promote wound healing but also achieve on-demand removal to reduce the pain of dressing change, especially for acute and chronic wound that required long-term, repeated dressing change [67,68]. Recently, Wen and colleagues [69] proposed a detachable wound dressing with asymmetric wettability. The dressing consisted of 3 layers, including inner layer of gelatin-giant salamander fiber for adhesion to the wound, the outer layer of nanofiber for overall mechanical support, and the middle cross-linked layer for connecting inner and outer layer. At room temperature, the molecular chains were more active to expose more functional groups, producing the strong adhesion between tissue and dressing. At low temperature, the molecular chains were arranged neatly, exposing fewer surface functional groups, resulting in reduced adhesion for easy separation from wound (Fig. 3D). Hydrogel had become the preferred material for wet dressing due to their strong hydrophilicity, good biocompatibility, and similarity to extracellular matrix [70]. However, its limited liquid absorption capacity tended to cause accumulation of secretion around wound. In recent years, self-pumping hydrogel dressing had attracted wide attention, which could quickly discharge excess wound exudate and reduce secondary injuries caused by frequent dressing replacement. Wang and colleagues [71] reported a self-pumping organohydrogel dressing consisting of a hydrophobic organogel layer, aligned hydrated channels, and a hydrophilic hydrogel layer. The asymmetric wettability structure and aligned hydrated channels work together to allow rapid, unidirectional discharge of viscous fluid from wound. What is more, the self-pumping dressing could attach to the dry skin around wound and easily separate from the newly formed wet tissue of the wound when dressing was changed, avoiding secondary injury (Fig. 3E).

### Sandwich structure

**Fig. 4.**
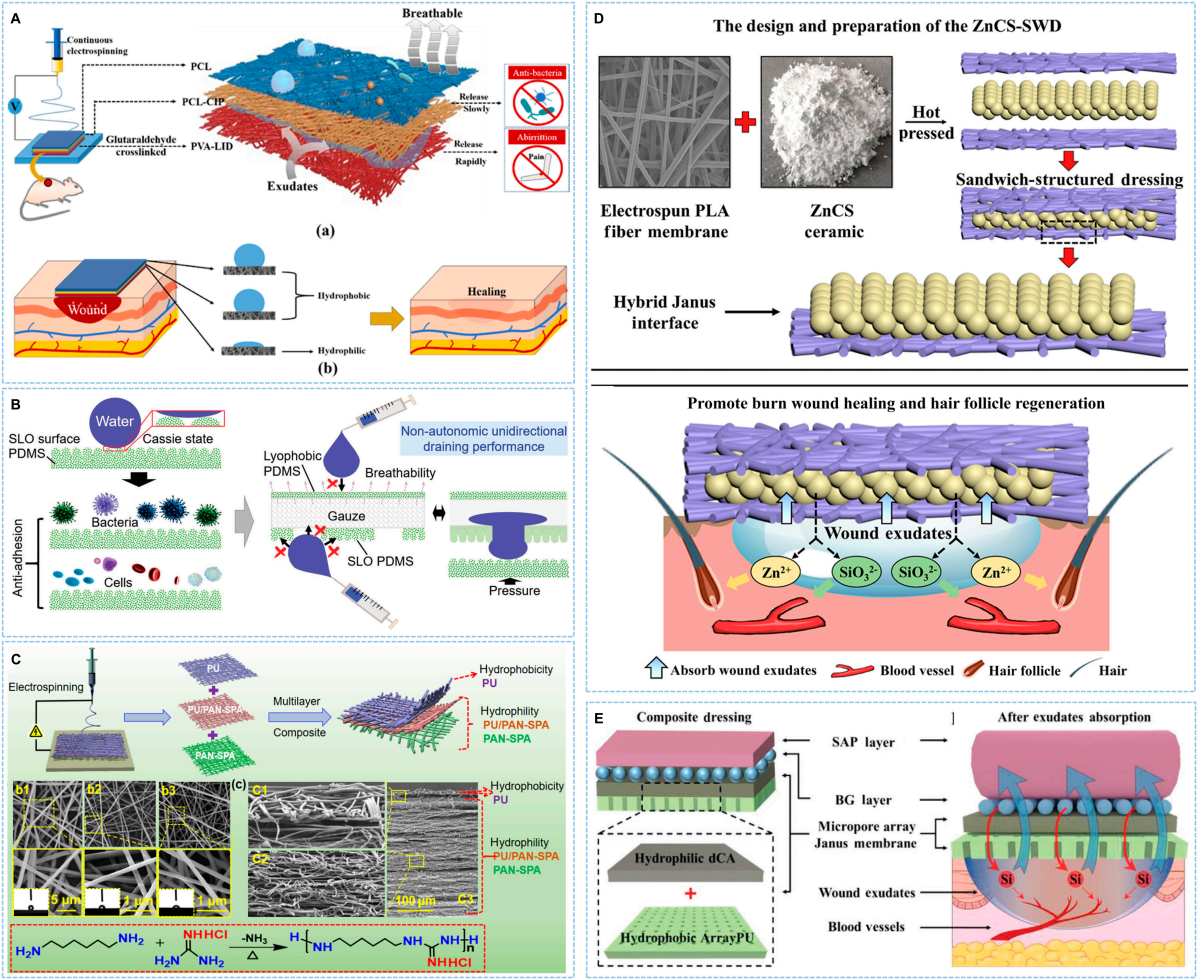
Asymmetric wettability dressing with sandwich structure. (A) Schematic diagram of the fabrication and application of trilayer asymmetric wettability dressing. Reproduced with permission [72]. Copyright 2024, KeAi. (B) Schematic illustration of the unidirectional drainage process of sandwich-structured wound dressing. Reproduced with permission [73]. Copyright 2022, Wiley. (C) Schematic representation about the preparation of unidirectional liquid transport dressing. Reproduced with permission [74]. Copyright 2021, Elsevier. (D) Preparation process of sandwich-structured dressing and its application in deep burn wound. Reproduced with permission [75]. Copyright 2021, KeAi. (E) Schematic representation of the construction of multifunctional composite dressing and the process of outward removal of wound exudate and return of bioactive ions. Reproduced with permission [76]. Copyright 2020, Wiley-VCH.

The sandwich-structured dressing usually consisted of 3 or more layers of material, which was formed by placing the core layer in the middle. The common preparation method was the layer stacking using electrospinning technology. Chen and colleagues [72] developed a 3-layer nanofiber membrane with asymmetric wettability. The outer hydrophobic PCL layer could isolate wound from the external environment, reduce water evaporation, and prevent microorganism invasion. The middle layer could inhibit bacterial growth, and the hydrophilic inner layer had the excellent water absorption ability. This dressing had an asymmetric wettability structure where the exudate was transported unidirectionally within the material and maintained between the bottom and middle layer without being contaminated by external environment (Fig. 4A). Hou and colleagues [73] presented a sandwich-structured wound dressing. The hydrophobic outer layer had unidirectional drainage and resistance to adhesion, and exudate that built up between wound and dressing was absorbed and extruded by intermediate gauze layer through microporous channels of the inner superhydrophobic layer, realizing the effective exudate management (Fig. 4B). Chen and colleagues [74] designed a 3-layer unidirectional water delivery dressing using a hydrophobic PU nanofiber film as the wound contact layer, which could effectively reduce the adhesion between wound and dressing. A hydrophilic nanofiber membrane with rich micropores was the outermost layer to isolate external bacteria. The middle hydrophilic layer was designed to absorb and pump exudate (Fig. 4C). Severe burn wound usually secreted large amounts of exudate, which was easy to infection and difficult to heal. Chang and colleagues [75] used hydrophilic zinc silicate bioceramics and hydrophobic polylactic acid to prepare asymmetric wound dressing with sandwich structure by hot pressing forming method. This unique organic/inorganic Janus dressing showed excellent exudate handling capabilities, creating a favorable environment for wound healing (Fig. 4D). To relieve edema and stimulate angiogenesis, Chang and colleagues [76] designed a multifunctional composite wound dressing. Different from the unidirectional liquid transport function of traditional Janus structure, the modified Janus membrane imbedded micro-pores into the hydrophobic PU layer to obtain bidirectional infusion capability. Moreover, a bioactive layer was placed above the modified Janus membrane to release bioactive ions after contacting with wound exudate, which then returned to wound bed through Janus membrane to promote angiogenesis and healing (Fig. 4E).

### Gradient structure

**Fig. 5.**
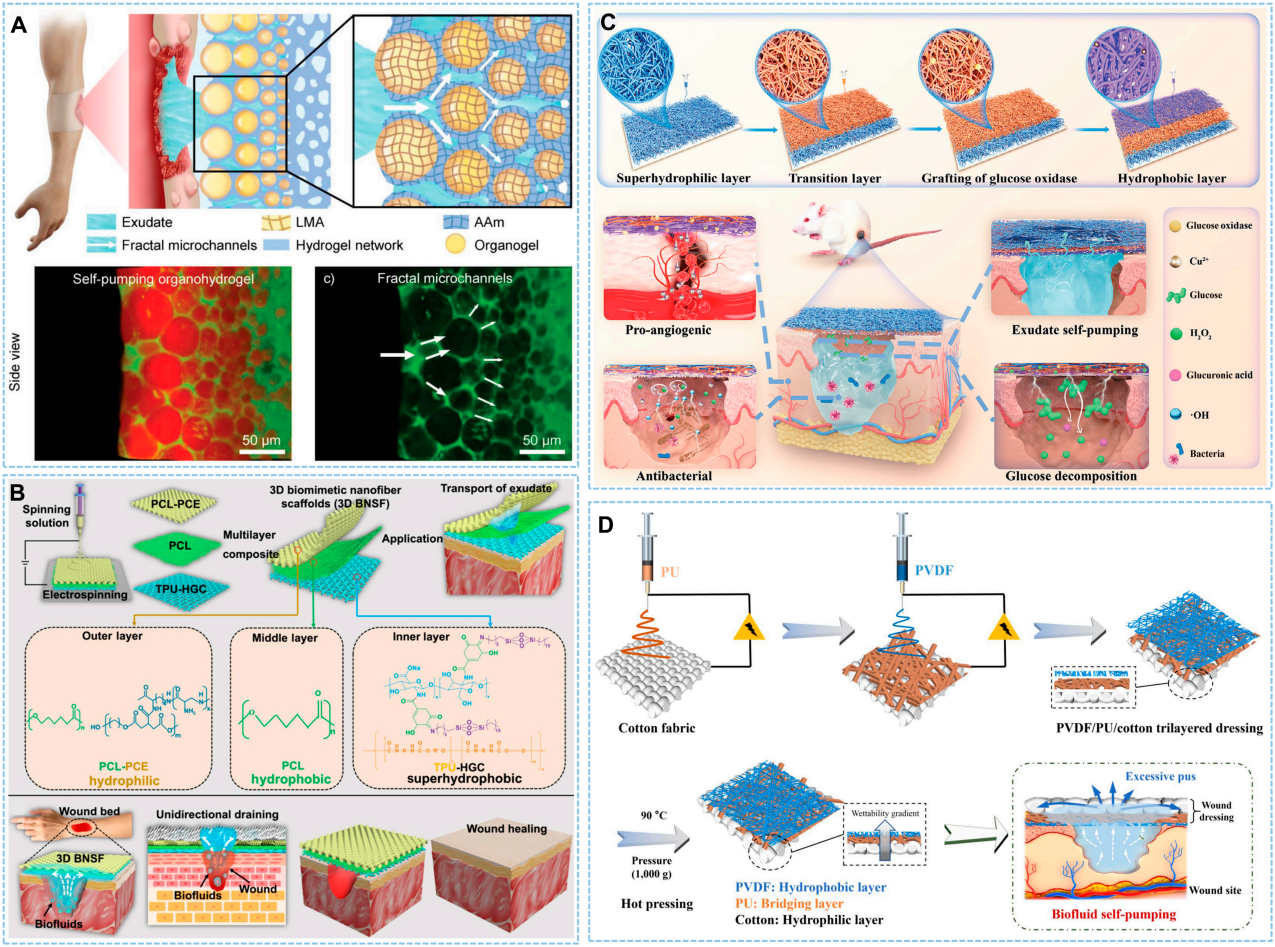
Gradient asymmetric wettability dressing. (A) Schematic illustration of the design of self-pumping organohydrogel dressing. Reproduced with permission [78]. Copyright 2023, Wiley-VCH. (B) Schematic diagram of the preparation of a 3D nanofiber scaffold and the process of fluid transport. Reproduced with permission [80]. Copyright 2024, Springer Nature. (C) Fabrication of wound dressing and the exudate drainage process. Reproduced with permission [81]. Copyright 2024, Wiley-VCH. (D) Schematic representation of the fabrication of trilayered dressing. Reproduced with permission [82]. Copyright 2022, Springer Nature.

Gradient wettability usually meant that the wettability of one or more materials changed in a certain direction. In a narrow sense, a material with gradient performance could be constructed by making the structure and components unevenly dispersed in the material, forming the gradient asymmetric wettability dressing [77]. Wang and colleagues [78] reported a self-pumping organic hydrogel dressing with hydrophilic micro-channels by emulsion polymerization, in which the organogel particles were mixed with polyacrylamide hydrogel and the particle size was reduced from outside to inside. Compared with pure hydrogel, the dressing can quickly discharge excessive exudate, and the efficiency was about 30 times higher (Fig. 5A).

In a broad sense, the gradient asymmetrical wettability dressing referred to the preparation of multilayers of dressing through several materials with different wettability to obtain the gradient wettability dressing [79]. Pang and colleagues [80] introduced a 3D nanofiber scaffold with wetting gradient by combining 3 different materials of superhydrophobic/hydrophobic/hydrophilic, which could better adapt to the shape and size of a particular wound to improve the problem of biofluid transfer slowly (Fig. 5B). To address the problem of limited drug loading and nonsustainable antimicrobial effect of some self-pumping dressings, Zhao and colleagues [81] developed a Janus membrane with gradient hydrophilicity property for fluid self-pumping, in which polyacrylonitrile (PAN) fiber was superhydrophilic layer, PU fiber was hydrophobic layer, and the intermediate transition layer was obtained by co-electrospinning PAN and PU. While wound exudate was pumped out, the glucose oxidase (GOx) and copper ions (Cu^2+^) in the Janus membrane could trigger a series of cascading reactions, producing bactericidal effect, promoting angiogenesis and wound healing (Fig. 5C). Gradient wettability materials had excellent liquid directional transport performance, but the compatibility between layers with different wettability was poor, and the weak interface bonding led to interlayer air gap, which was not conducive to continuous liquid pumping. Recently, Wu and colleagues [82] reported a 3-layer structured fiber dressing with gradient wettability prepared based on electrospinning technology, which achieved a stable interlayer composite and an efficient single-guide solution, promoting wound healing. By electrospinning PU and polyvinylidene fluoride (PVDF) sequentially on the surface of hydrophilic cotton fabric, the cotton/PU/PVDF composite was made stable under hot pressing condition by using the hot melt bonding effect of PU. The dressing had good underwater adhesive stability and high efficiency of single-guide fluid, which had great potential in the exudate management of severe wound (Fig. 5D).

## Outlook and Summary

In the process of wound healing, timely and effective monitoring of wound status was of great significance to reduce infection and accelerate wound healing. However, the current judgment of wound status mainly depended on the experience of the medical staff, which could easily lead to bias or error. Moreover, frequent hospital visit by patients in need of treatment increased their physical and financial stress [83].

**Fig. 6.**
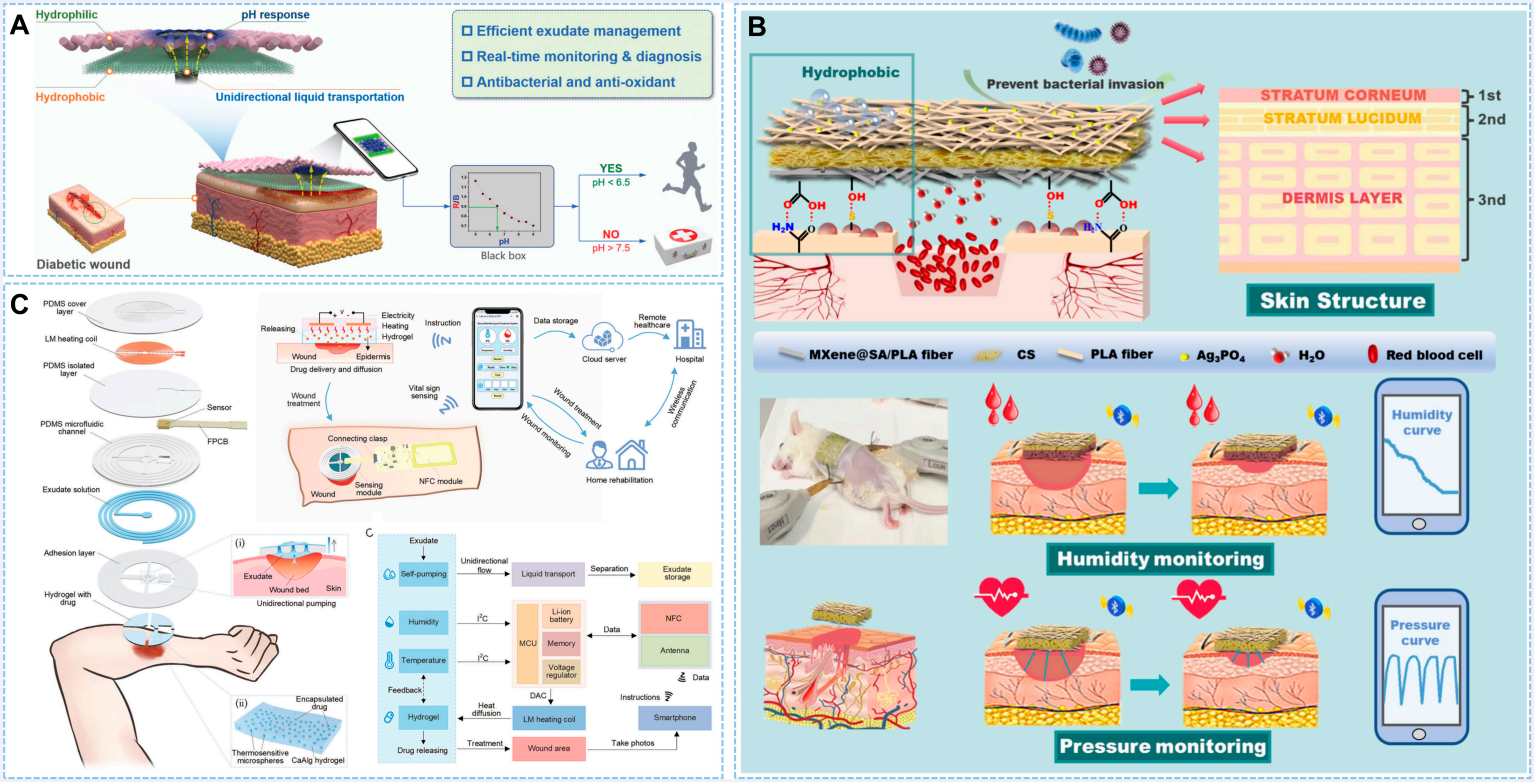
Asymmetric wettability dressing with wound monitoring. (A) Schematic representation of the dressing for exudate drainage and diabetic wound healing monitoring. Reproduced with permission [92]. Copyright 2023, Wiley-VCH. (B) Schematic illustration of the dressing structure and the monitoring process through humidity and pressure signals. Reproduced with permission [93]. Copyright 2024, Springer Nature. (C) Schematic diagram of the design of wound patch and wound status monitoring. Reproduced with permission [94]. Copyright 2023, Wiley-VCH.

In the last decade, smart wound dressings had emerged endlessly, including stimuli-response wound dressings [6], self-removal wound dressings [84], and monitoring wound dressings [85–88], which could communicate with wound through built-in sensors and/or smart materials to sense the wound status and provide feedback, effectively helping wound management [89–91]. Sun and colleagues [92] proposed a smart dressing with the capabilities of rapid dehumidification, non-adhesion, pH response, and antimicrobial for wound status monitoring and exudate management. At different stages of wound healing, the dressing could produce different colors in response to the pH of exudate, allowing real-time monitoring of healing process, and removing excess exudate to weaken the wet adhesion. Compared with commercial gauze, the dressing had higher healing promotion rate (Fig. 6A). Inspired by human skin, Wang’s group [93] developed a smart wound dressing with an asymmetrical wettability and 3-layer structure, which could optimize exudate management and accelerate wound healing, and humidity and pressure at the wound site could be monitored in real time through Bluetooth wireless connection (Fig. 6B). A new generation of smart wound dressings based on flexible and stretchable sensors enabled bedside diagnosis and on-demand drug release in response to wound condition. Ren’s group [94] developed an intelligent wound dressing, which could achieve unidirectional pumping and on-demand drug delivery based on temperature and humidity information (Fig. 6C). It could be seen that a smart wound dressing that could diagnose the stage of wound healing, suggest the timing of dressing change, and effectively simplify daily care was highly significant for patients and medical staff.

In this review, we summarized the latest progress of 3 kinds of asymmetric wettability wound dressings in exudate management, including Janus structure, sandwich structure, and gradient structure. The most common Janus structural dressing among asymmetric wettability dressings was highlighted from 2 aspects: single-layer modified Janus structure and double-layer Janus structure. The challenges faced by asymmetric wettability wound dressings were discussed, and the demand and developing trend of smart wound dressings in this field were prospected. We presented the wound exudate management dressing in an illustrated way, providing a comprehensive perspective on the development of wound dressing.

## Data Availability

No data were used for the research described in the article.
